# Inflammatory bowel diseases in Tamil Nadu: A survey of demographics, clinical profile, and practices

**DOI:** 10.1002/jgh3.12673

**Published:** 2021-11-05

**Authors:** Rohan V Yewale, Kartik Natarajan, Jeyaraj Ubal Dhus, Sarojini Ashok Parameswaran, Kallipatti Ramaswamy Palaniswamy, Doraisamy Babu Vinish, Aravindh Somasundaram, Arulraj Ramakrishnan, Sibithooran Karmegam, Ramaswamy Saraswathy Arun, Ujjani Shankaraiah Manmohan, Balakrishnan Mahadevan, Baskkaran Harri Prasad, Toguluva Seshadri Chandrasekar, Bollu Janakan Gokul, Amit Dutta, Anjilivelil Joseph Joseph, Jayanthi Venkatraman, Panchapakesan Ganesh, Subramanyam Shanmuganathan, Palaniappan L Alagammai, Ramalingam Ramasubramanian, Leelakrishnan Venkatakrishnan, Ramamoorthi Ganesan, Arunachalam Chandrasekaran Arun, Sankaranarayanan Srinivas, Mariappan Kannan, M Shanmugam Revathy, Malathi Sathiyasekaran, Arulprakash Sarangapani, Natrajan Rajesh, Velusamy Arulselvan, Arumugam Aravind, Karunakaran Premkumar, Sampath Kavitha, Hema Vijayalakshmi Varadarajulu, Murugesan Manimaran, Pandurangan Basumani, Ananthavadivelu Murali, Balakrishnan Siddartha Ramakrishna

**Affiliations:** ^1^ SRM Institutes for Medical Science Chennai India; ^2^ Apollo Hospital Chennai India; ^3^ Kovai Medical Center and Hospital Coimbatore India; ^4^ Madras Medical Mission Hospital Chennai India; ^5^ Gleneagles Global Health City Chennai India; ^6^ GEM Hospital Coimbatore India; ^7^ MedIndia Hospitals Chennai India; ^8^ Christian Medical College Vellore India; ^9^ Sri Ramachandra Institute of Higher Education & Research Chennai India; ^10^ Meenakshi Mission Hospital Madurai India; ^11^ Thoothukudi Medical College Thoothukudi India; ^12^ PSG Institute of Medical Science & Research Coimbatore India; ^13^ Thanjavur Medical College Thanjavur India; ^14^ Vellamal Medical College Hospital Madurai India; ^15^ Kanchi Kamakoti Childs Trust Hospital Chennai India; ^16^ Madurai Medical College Madurai India; ^17^ Stanley Medical College Chennai India; ^18^ MGM Healthcare Chennai India; ^19^ Sri Gokulam Hospital Salem India; ^20^ Coimbatore Medical College Coimbatore India; ^21^ DDHD, Government Peripheral Hospital Chennai India; ^22^ Madras Medical College Chennai India; ^23^ Governmentt Royapettah Hospital Chennai India; ^24^ Sri Chakra Multispecialty Hospital Chennai India; ^25^ GEM Hospital Chennai India; ^26^ Dr. Rela Institute and Medical Centre Chennai India; ^27^ MIOT International Chennai India

**Keywords:** Crohn's disease, epidemiology, pediatric inflammatory bowel disease, ulcerative colitis

## Abstract

**Background:**

Inflammatory bowel disease (IBD) is increasingly diagnosed in South Asia. This survey by the Tamil Nadu Chapter of the Indian Society of Gastroenterology (TNISG) documents the demography, clinical profile, and therapeutic practices related to IBD in Tamil Nadu.

**Methods:**

TNISG members from 32 institutions completed an online cross‐sectional questionnaire on IBD patients from March 2020 to January 2021.

**Results:**

Of 1295 adult IBD patients, 654 had Crohn's disease (CD), 499 ulcerative colitis (UC), and 42 IBD‐unclassified (IBD‐U). CD and UC showed a unimodal age distribution. A total of 55% were graduates or postgraduates. A positive family history was noted in 30, other risk factors were uncommon. In CD, the pattern of involvement was ileocolonic (42.8%), ileal (34.7%), colonic (18.9%), and upper gastrointestinal (3.5%); while in UC, disease was characterized as extensive (44.9%), left‐sided (41.7%), or proctitis (13.4%). Perineal disease, perianal fistulae, and bowel obstruction were noted in 4.3, 14.0, and 23.5%, respectively, of CD. The most widely used drugs were mesalamine, azathioprine, and corticosteroids. Surgery was undertaken in 141 patients with CD and 23 patients with UC. Of the 138 patients with pediatric IBD (≤16 years), 23 were characterized as very early onset IBD (VEO‐IBD), 27 as early‐onset, and 88 as adolescent IBD. VEO‐IBD were more likely to have a positive family history of IBD and were more likely to have perineal disease and to have the IBD‐U phenotype. Among pediatric IBD patients, corticosteroids, mesalamine, and azathioprine were the most commonly used medications, while 25 pediatric patients received biologics.

**Conclusion:**

This study provides important information on demography, clinical profile, and treatment practices of IBD in India.

## Graphical abstract

Regional differences in inflammatory bowel disease (IBD) profile inform our understanding of the disease as well as prevention, and health planning. This study provides a snapshot of IBD presentation and practices in India at a time when the country is in the second of four phases of IBD transition. The apparently mild nature of IBD, the consequent widespread use of mesalamine, and the association of Crohn's disease with higher levels of education, compared to ulcerative colitis, are of notable interest.

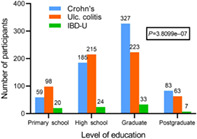

## Introduction

The Indian subcontinent has witnessed a remarkable rise in incidence and prevalence of inflammatory bowel disease (IBD) over the past few decades.^1^, ^2^, ^3^, ^4^, ^5^ India has the highest number of IBD cases among South and South East Asian countries owing to its huge population as well as the rising disease burden.^6^ Although the genetic architecture of IBD in India and the West exhibits both similarities as well as differences, the overall genetic risk in Indians is similar to the Western population.^2^ This has been well demonstrated by studies depicting similar disease susceptibility in second‐generation Indian immigrants living in high prevalence areas for IBD.^7^ A recent population‐based study conducted in the United States with an objective of estimating the prevalence of IBD in patients of different ethnicities concluded that residents of Indian ancestry had a greater risk for all types of IBD than other American populations.^8^ Consistent with this trend, the changing epidemiology of IBD on the global map has been referred to as a prime example of “the third epidemiological transition.”^9^

The rise in disease burden has prompted the establishment of various regional and national IBD disease registries over the past few years. The Indian Society of Gastroenterology (ISG) Task Force on IBD has previously conducted a multi‐center survey and collated data on the clinical spectrum of IBD from five zones (North, Central, West, East, and South) across the country in the year 2012.^10^ Subsequently, there have been multiple publications on numerous epidemiological aspects of IBD from different regions across the country.^11^, ^12^, ^13^, ^14^ Most recently, the Colitis and Crohn's Foundation (CCF), India, launched a nationwide, multicentric prospective IBD registry involving referral centers from the four geographical zones of India.^15^ A caveat in the present national IBD registries is that the respective zonal data does not entirely represent the individual states in the zone. Despite being identified as one of the major health capitals of India, there is paucity of epidemiological data on IBD from the state of Tamil Nadu. With this background, the Tamil Nadu Chapter of Indian Society of Gastroenterology (TNISG) decided to create an IBD Tamil Nadu Consortium (IBD‐TNC) in January 2020 with the primary objective of assembling data pertinent to IBD from multiple caregivers across Tamil Nadu. The purpose of this state‐wide registry was to obtain a representative picture of the current demography, clinical profile, and management practices in the state with respect to IBD. The ultimate goal of the IBD‐TNC was to formulate a registry with “real world data,” which would be accessible to clinicians for future research and would contribute to the national IBD database.

## Methods

The core committee of the IBD‐TNC drafted a cross‐sectional anonymized data collection questionnaire, which was uploaded on the TN‐ISG web portal for online data entry. The questionnaire consisted of basic questions, which dealt with the demography, environmental risk factors, family history, clinical spectrum, and prescribing practices in IBD to be filled by the participating member of the IBD‐TNC. IBD‐TNC members received initial notification and periodic reminders from the Secretary TN‐ISG. Participation in the survey involved uploading of IBD patient data without individual patient identifiers onto the electronic database maintained by the TN‐ISG secretariat. Participating physicians were requested to ensure completeness in filling the questionnaire. All the respondents were qualified medical gastroenterologists working in public or private sector settings across Tamil Nadu. The time frame for the study was set from March 2020 to January 2021. The study proposal was approved by the Ethics Committee of the SRM Institutes for Medical Science, Chennai (approval number SIMS/IEC/Other/03/2020). All study practices were in conformance with the Helsinki declaration of 1975, as revised in 2000 and 2008. At the end of the study period, the data were transferred into Excel sheets by a data‐entry operator and provided to RVY and KN for analysis. The diagnosis was marked as ulcerative colitis (UC), Crohn's disease (CD), or unclassified IBD (IBD‐U).

### Statistical analysis

Continuous data were expressed as mean (standard deviation) or median (interquartile range, IQR) based on their distribution. Categorical data were represented as frequencies (number of cases) and percentages as appropriate. All the study variables were compared between the three categories of IBD, namely UC, CD, and IBD‐U. Categorical data were compared between the groups using the Chi‐square test. Quantitative characteristics were compared using the *t*‐test or Mann–Whitney *U* test as appropriate. A probability value (P‐value) less than 0.05 was considered statistically significant. An interim analysis of the study was presented by the core committee to the members of the TN‐ISG during the state mid‐term conference in the year 2020. Statistical calculations were performed using SPSS version 25.0 (IBM Corp, Armonk, NY, USA).

## Results

Thirty‐two health institutions across Tamil Nadu, from both government as well as private sector, participated in the survey (Table 1). Forty‐eight entries that were either incomplete or duplicate were rejected. Data pertaining to the remaining 1433 IBD patients (739 CD [51.6%], 637 UC [44.4%], and 57 IBD‐U [4%]) was available for analysis.

**Table 1:** List of participating institutions

S. no	Institution
1	SIMS Hospital, Chennai
2	Apollo Hospital, Chennai
3	Kovai Medical College, Chennai
4	MMM Hospital, Chennai
5	Global Hospital, Chennai
6	GEM Hospital, Coimbatore
7	Medindia Hospital, Chennai
8	Christian Medical College, Vellore
9	SRIHER, Chennai
10	Meenakshi Mission Hospital, Madurai
11	Thoothukudi Medical College, Thoothukudi
12	PSG Medical College, Coimbatore
13	Thanjavur Medical College, Thanjavur
14	Velammal Medical College, Madurai
15	Kanchi Kamakoti Child Trust Hospital, Chennai
16	Madurai Medical College
17	Stanley Hospital, Chennai
18	MGM Hospital, Chennai
19	Shri Gokulam Hospital, Salem
20	Coimbatore Medical College, Coimbatore
21	Digestive Diseases & Health Department, Chennai
22	Madras Medical College, Chennai
23	Government Royapettah Hospital, Chennai
24	Sri Chakra Hospital
25	GEM Chennai Hospital
26	Rela Institute of Medical Sciences, Chennai
27	MIOT Hospital, Chennai
28	Apollo Hospitals Madurai
29	Government Mohan Kumaramangalam Medical College, Salem
30	St. Isabel's Hospital, Chennai
31	Tirunelveli Medical College
32	Fortis Malar Hospital

### Demographics

**Figure 1:**
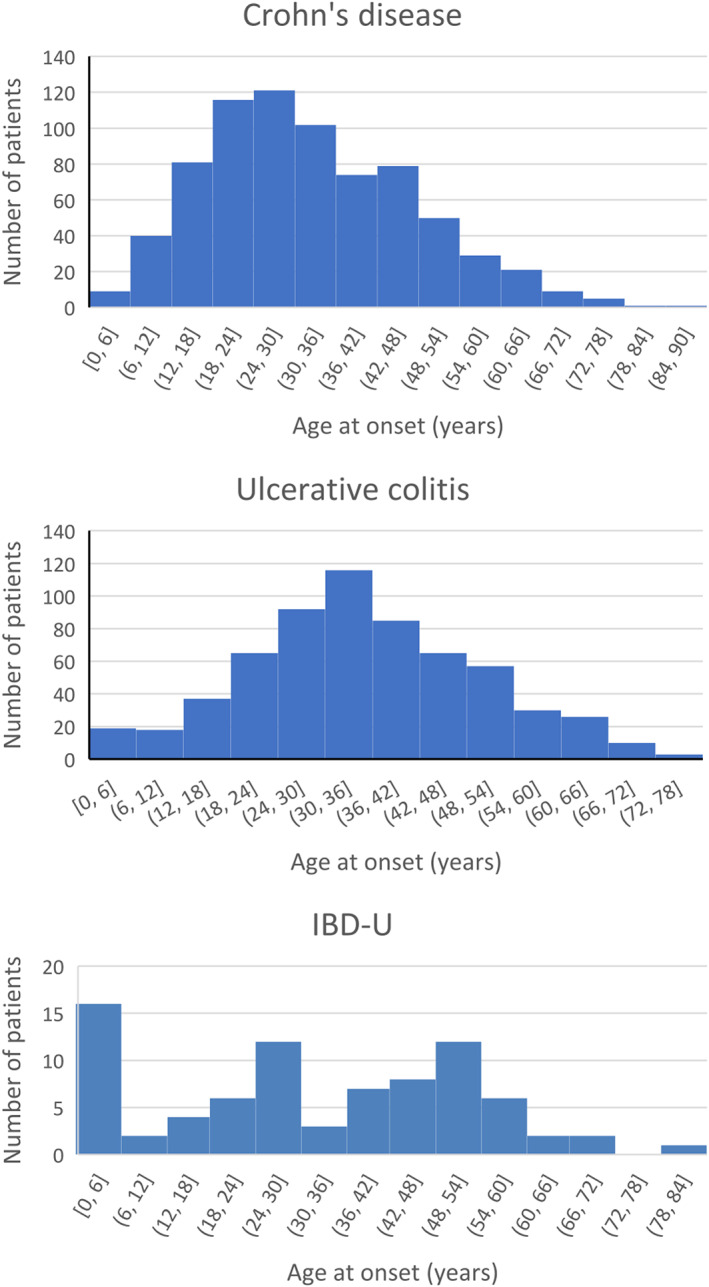
Age distribution of patients with CD, UC, and IBD‐U. The X‐axis shows 6‐year age intervals, and the Y‐axis shows the number of patients in each age interval.

The median (range) age of the patients at the time of IBD diagnosis was 33 years (2 months to 89 years). The median age was 31 years for CD patients, 35 years for UC patients, and 31 years for IBD‐U patients. Figure 1 shows the distribution of age at diagnosis in this cohort of patients. Both CD and UC showed a unimodal distribution with a slightly lower age at diagnosis for CD patients (24–30 years) compared to UC patients (30–36). IBD‐U showed a multimodal distribution. CD showed a male preponderance (M:F = 1.33:1); UC (M:F = 1.06:1) did not show any sex predilection; and IBD‐U showed a female preponderance (M:F = 0.76:1). Median (range) duration of symptoms prior to diagnosis was 12 (0–348), 24 (1–432), and 6 (1–144) months, respectively, for CD, UC, and IBD‐U.

### Pediatric IBD

For the purposes of this study, we classified patients of 16 years or younger as pediatric IBD. This age cut‐off is consonant with the Porto criteria^16^ as well as the Montreal classification^17^ in which the A1 group is 16 years old or less. In the present survey, 138 patients belonged to the pediatric age group. Table 2 shows their distribution across the three different phenotypes. It is interesting that there were patients both with the very early onset IBD (VEO‐IBD) phenotype and the early onset IBD (EO‐IBD) phenotype. Table 2 lists the characteristics of these patients, including their symptomatology, the duration of symptoms prior to diagnosis, the extent of disease, extraintestinal manifestations (EIM), and treatment offered. It is interesting to note that the majority of children with VEO‐IBD had the IBD‐U phenotype followed by the UC phenotype while the CD phenotype was the least common. On the other hand, the CD phenotype was the most common both in EO‐IBD and adolescent IBD, with the dominance of CD being more in adolescent IBD. Median duration of the disease was shorter in VEO‐IBD as compared to EO‐IBD and adolescent IBD. Diarrhea followed by blood in the stool was the most common symptom in VEO‐IBD, whereas abdominal pain and diarrhea were the most common symptoms in older children with IBD. Family history of another family member with IBD was present in 5/23 (21.7%) of VEO‐IBD, compared to 2/27 (7.4%) and 5/88 (5.7%) adolescent IBD. Diagnosis was achieved using a combination of endoscopic and radiological imaging, with upper gastrointestinal endoscopy being more commonly used in VEO‐IBD while colonoscopy was more commonly used in older children with IBD. MR enterography was not widely used, possibly because of lack of wide availability. Perianal and perineal disease were the most common EIM of IBD in these children. On the whole, EIM were not common. Corticosteroids, mesalamine, and azathioprine were the most commonly used drugs in these patients. Overall 25 pediatric IBD patients received biologics, including 18 patients in the adolescent IBD group. Interestingly, 10 of 23 (43.5%) VEO‐IBD had a history of appendectomy.

**Table 2:** Characteristics of 138 pediatric IBD patients

	VEO‐IBD	EO‐IBD	Adol‐IBD
*n*	23	27	88
Phenotype			
Crohn's disease	4	16	65
Ulcerative colitis	6	9	23
IBD‐Undifferentiated	13	2	0
Male:female	13:10	12:15	41:50
Median duration, months	3	6	12
Symptoms			
Abdominal pain	12	23	73
Diarrhea	19	18	73
Blood in stool	17	15	45
Weight loss	16	16	48
Anorexia	11	16	33
Fever	15	7	16
Family history	5	2	5
Evaluation			
Upper GI endoscopy	20	15	44
Colonoscopy	17	27	89
CT enterography	8	10	41
MR enterography	5	1	9
Video capsule endoscopy	4	0	2
Enteroscopy	7	0	0
Extraintestinal manifestations			
Arthritis	2	0	5
Erythema nodosum	2	0	6
Pyoderma gangrenosum	2	0	1
Episcleritis	2	0	1
Uveitis	1	0	0
Primary sclerosing cholangitis	1	0	0
Perineal disease	8	0	9
Behavior of the disease			
Perianal fistula	4	0	22
Urinary fistula	1	0	0
Intestinal obstruction	2	1	16
Disease extent			
Crohn's—L1/L2/L3/L4	1/3/0/0	6/2/8/0	20/12/33/0
Ulcerative colitis E1/E2/E3	0/3/3	2/2/5	0/9/14
Risk factors			
Appendectomy	10 (1 CD, 3 UC)	0	7 (5 CD)
Oral contraceptives	0	0	0
NSAID use	0	0	0
Smoker	0	0	0
Treatment			
Mesalamine tablet	5	17	58
Mesalamine granules	9	8	22
Prednisolone	13	18	65
Budesonide	3	2	4
Azathioprine	8	13	54
Methotrexate	1	0	0
Infliximab	5	1	9
Adalimumab	0	1	9

Very early‐onset IBD (VEO‐IBD, 0–5 years), early‐onset IBD (EO‐IBD, 6–10 years), and adolescent IBD (Adol_IBD, 11–16 years). NSAID, non steroidal anti inflamatory drug.

### Adult IBD

#### Clinical features

There was a male preponderance among patients with CD, while UC showed an equal sex ratio, and IBD‐U was more common in females (Table 3). Symptoms in these patients included abdominal pain, diarrhea, fever, blood in stools, weight loss, anorexia, and fever. Abdominal pain was the most common symptoms in patients with CD, while diarrhea with blood was the most common symptom in patients with UC (Table 3). Colonoscopy and biopsy was the most common diagnostic modality in these patients. Upper gastrointestinal (GI) endoscopy was done only in about a third of patients. Small bowel imaging with CT enterography was done more often in patients with CD compared to UC, while MR enterography was done in a much smaller number. Capsule enteroscopy and push enteroscopy were done in a few patients mostly those with CD (Table 3).

**Table 3:** Characteristics of adult IBD patients surveyed in the present study

	Crohn's disease	Ulcerative colitis	IBD‐undifferentiated
*n*	654	599	42
Male:female	378:276	305:294	30:54
Symptoms			
Abdominal pain	565	317	43
Diarrhea	444	441	52
Blood in stool	150	497	24
Weight loss	392	207	27
Anorexia	196	103	19
Fever	64	47	3
Evaluation			
Upper GI endoscopy	280	161	27
Colonoscopy	615	579	72
CT enterography	358	83	20
MR enterography	34	4	2
Video capsule endoscopy	9	3	2
Enteroscopy	23	0	1
Local complications			
Perineal disease	28	1	1
Perianal fistula	92	14	1
Vesical fistula	3	1	0
Bowel obstruction	154	11	2
Disease extent	L1–227	E1–80	
	L2–124	E2–250	
	L3–280	E3–269	
	L4–23		
Treatment			
Mesalamine tablet	422	539	66
Mesalamine granules	116	24	2
Prednisolone	374	376	42
Budesonide	103	18	7
Azathioprine	404	207	20
Methotrexate	8	1	1
Infliximab	27	3	2
Adalimumab	57	11	5
Surgery	141	23	12

#### Associations

**Figure 2:**
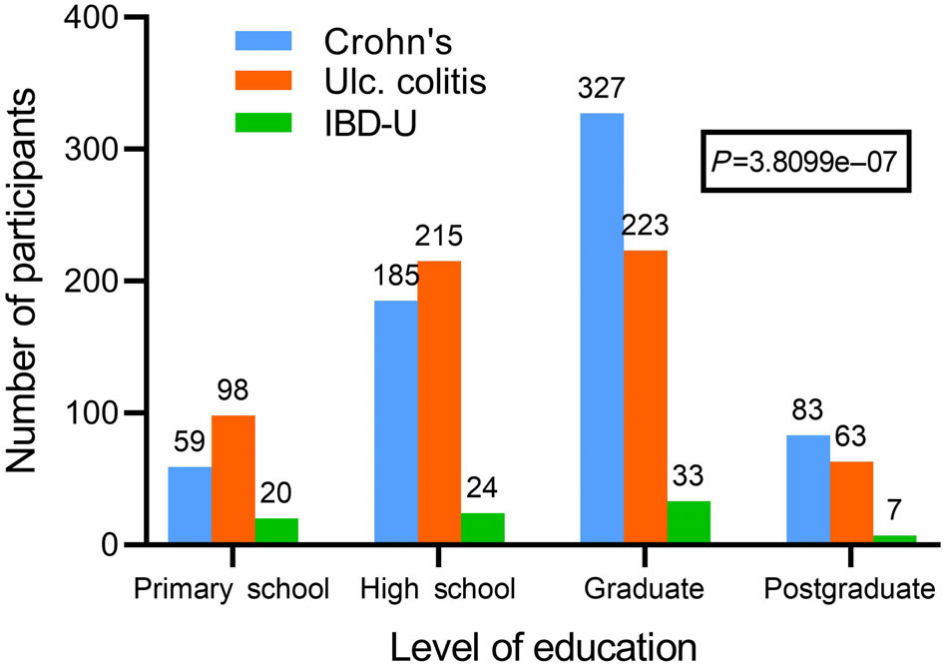
Number of individuals in each disease group classified by highest education level.

The majority of the adult IBD patients (55%) were either graduates or postgraduates (Fig. 2), while 13.2% had only been educated up to primary school level. The proportion of CD patients (62.6%) who were graduates and postgraduates was significantly greater than that among UC patients (47.7%) (Fig. 2). A history of appendectomy was noted in 3.5% of adult CD patients compared to 1.6% of adult UC patients (Table 4). A history of use of oral contraceptives or of nonsteroidal analgesic drugs was obtained only in a minority of patients, being marginally greater in UC patients compared to CD patients (Table 4). Among the adult IBD patients, a positive family history was noted only in 30 patients (2.25%), and did not vary significantly between CD (2.29%) and UC (2.00%) (Table 4).

**Table 4:** Presence of known risk factors in adult IBD patients

Sr. no	Risk factor	CD (*n* = 654)	UC (*n* = 599)	IBD‐U (*n* = 42)
1	Appendectomy	23	10	3
2	OCP use	6	9	0
3	NSAID use	1	7	1
4	Smoking	28	21	12
5	Family h/o IBD	15	12	3

#### Extent and behavior of disease

Among patients with CD, the most common pattern of intestinal involvement noted was ileocolonic disease (42.8%) followed by ileal disease (34.7%) and isolated colonic disease (18.9%). Isolated upper gastrointestinal disease (esophagus, stomach, and/or duodenum) was noted in 23 patients (3.5%). Among UC patients, pancolitis was the most common category (44.9%), followed by left‐sided colitis (41.7%) and proctitis (13.4%). Perineal disease was noted in 28 patients with CD, and in 1 patient each with UC and IBD‐U. Perianal fistulae were common in CD (14.0%) compared to UC (2.3%) or IBD‐U (2.4%). Bowel obstruction was common in patients with CD (23.5%). Colonic obstruction was noted in 11 (1.8%) of UC patients and 2 (4.8%) of IBD‐U patients. Internal fistulae were apparently uncommon, with a total of four vesical fistulae being reported (Table 3). Table 5 shows the overall extent and behavior of CD in this cohort as per the Montreal classification.^17^ Notably, obstruction was associated with ileocecal or isolated ileal disease. In adult CD patients, perianal disease was noted with all locations of disease.

**Table 5:** Behavior and extent of Crohn's disease and ulcerative colitis as per the Montreal classification^17^

	A1	A2	A3
L1	B1 19; B2 8; B3 0; p 7	B1 89; B2 46: B3 0; p 9	B1 69; B2 23; B3 0; p 3
L2	B1 17; B2 0; B3 0; p 3	B1 72; B2 17; B3 0; p 25	B1 34; B2 1; B3 0; p 6
L3	B1 31; B2 9; B3 1; p 16	B1 205; B2 29; B3 2; p 36	B1 72; B2 18; B3; p 10
L4	(0)	B1 17	B1 6

#### Histology

Granulomas were found on mucosal biopsies in 268 of 654 (41.0%) patients with CD. Other histologic findings reported on mucosal biopsies in these patients included cryptitis (349 patients), crypt abscesses (221 patients) crypt distortion (255 patients) and mucosal inflammation (502 patients). The histological findings reported on colonic mucosal biopsies from patients with UC included lamina propria inflammation (496), cryptitis (431), crypt distortion (402), crypt abscesses (311), and granulomas (22). In IBD‐U patients, findings reported on mucosal biopsy included mucosal inflammation (68), crypt distortion (29), cryptitis (28), and crypt abscesses (25). Interestingly, 20 of 84 (23.8%) IBD‐U patients were reported to have granulomas on mucosal biopsy.

#### Extraintestinal manifestations

Arthralgia was the most common EIM noted in 169 patients, while arthritis (61 patients) and episcleritis (12 patients) were the next most common numerically. Pyoderma gangrenosum and erythema nodosum were infrequent, the former being more common in UC compared to CD, while primary sclerosing cholangitis was noted exclusively in UC, affecting seven patients in all (Table 6).

**Table 6:** Extraintestinal manifestations in the adult cohort of IBD patients

	Crohn's disease	Ulcerative colitis	IBD‐undifferentiated
Arthralgia	69	89	11
Arthritis	35	24	2
Erythema nodosum	3	3	1
Pyoderma gangrenosum	3	7	0
Episcleritis	7	4	1
Uveitis	0	0	1
Primary sclerosing cholangitis	0	7	0

#### Treatment and prescribing pattern

Mesalamine was the most widely prescribed medication in patients with CD, UC, and IBD‐U (Table 3). More than 82% of patients with CD received oral mesalamine, over 64% in tablet form, and another 18% in granule form. Five hundred and sixty‐three (94%) UC patients received oral mesalamine as either tablets or granules. One hundred and fifty‐two UC patients received topical mesalamine as suppository or enema. Similar proportions of CD and UC patients received oral prednisolone and intravenous hydrocortisone. Oral budesonide was more frequently used in CD while rectal steroids were used predominantly in UC patients. Nearly 62% of CD patients were given azathioprine, while only 34.5% of UC patients were given this immunomodulator. A very small number of CD patients received methotrexate. Ninety‐eight of the adult IBD patients had received biologics, with the numbers being greater for CD (84 patients, 12.8%) compared to UC (14 patients, 2.3%). Adalimumab was preferred to infliximab. One hundred and forty‐one CD patients underwent surgery compared to 23 UC patients.

## Discussion

IBD is a global disease and its evolution can be stratified into four epidemiological stages: Emergence, acceleration in incidence, compounding prevalence, and prevalence equilibrium.^18^ In the present era, India and other South East Asian countries lie in the “emergence” stage of global evolution. The glaring rise in incidence and prevalence of IBD in newly industrialized countries such as India has paralleled the westernization of cultures and societies in these regions.^7^, ^19^, ^20^, ^21^ The present study provides a cross‐sectional analysis of IBD as seen in Tamil Nadu in South India at the present stage, and will be useful for future comparisons as we progress along the epidemiological stages of the disease. In addition, the study provides the first large set of data on pediatric IBD in India, including phenotypes, family history, and management practices.

The CCF India registry reported a North–South divide in the type of IBD with preponderance of CD in South India.^15^ The present study did not find a marked predominance of CD over UC, with the ratio being only 1.16:1, and was consistent with the report of the nation‐wide ISG Task Force survey conducted in the year 2012.^10^ Both UC and CD showed a bimodal age distribution, consistent with the CCF India registry data, and contradictory to the bimodal age distribution reported in the West.^15^, ^22^

Pediatric IBD is reported to constitute about 25% of total IBD.^23^, ^24^, ^25^ In multi‐ethnic Asian cohorts of pediatric IBD, there appears to be an over‐representation of Indian ethnicity,^26^ suggesting that the genetic determinants for pediatric IBD are present in the Indian population. In the present study, pediatric IBD constituted nearly 10% of all IBD. In an earlier single‐center study in South India, 34 cases of pediatric IBD were reported to constitute 7% of all new cases of IBD seen annually.^27^ A questionnaire survey of 221 children with IBD in India used an age cut‐off of 18 years and concluded that pediatric IBD resembled adult‐onset IBD.^28^ Our study used a cut‐off age of 16 years for pediatric IBD as this is consistent with Porto criteria and Montreal criteria for IBD phenotype. Further, we have subdivided pediatric IBD into VEO‐IBD (where the genetic influence is quite strong), EO‐IBD, and adolescent IBD. Of interest, VEO‐IBD was phenotypically often unclassified and presented with diarrhea or rectal bleeding, while EO‐IBD and adolescent IBD were more like CD and presented with abdominal pain and diarrhea. Fifty‐six percent (13/23) of the VEO‐IBD patients in this study had an IBD‐U phenotype compared to 30% in a recent study from India.^29^ Perineal and perianal disease occurred in about 52% of VEO‐IBD patients, indicating the aggressive nature of the disease. A positive family history was present in 21.7% of VEO‐IBD. Steroids, mesalamine, azathioprine, and biologics were the main modalities of therapy in this group. Overall, 25 patients with pediatric IBD (18.1%) received biologics as treatment compared to 8.1% of adult patients. Interestingly, appendectomy had been done in 10 of 23 (43.4%) VEO‐IBD patients. This high rate probably reflects the initial presentation of VEO‐IBD to pediatric surgeons rather than pediatric gastroenterologists, leading to the exploration of the abdomen with a diagnosis of appendicitis. An increased awareness, particularly with increasing availability of good imaging, may reduce the appendectomy rate in this group of patients.

A familial aggregation of IBD has been reported in 8–14% of patients from the West.^30^, ^31^ In the present study, a family history of IBD was present in 8.7% of pediatric IBD compared to 2.3% of adult IBD. Previous reports from India have suggested that 3–4.25% of IBD patients have another family member affected with IBD.^10^, ^32^, ^33^ As reported in the nationwide ISG survey,^10^ a history of appendectomy was more common in patients with CD rather than UC, and smoking and oral contraceptive pill consumption did not differ between CD and UC.

The overall clinical profile of the different phenotypes of IBD in the present study was similar to that reported by previous Indian epidemiological studies.^10^, ^13^ A few patients (14) with UC reported a history of perianal fistula in the past. While perianal fistulae are generally associated with CD or IBD‐U, fistulae, and abscesses may occur in a small proportion of patients with UC.^34^, ^35^ A sizable proportion of patients in the present study had some form of EIM. The present study provides significant detail about the phenotypic characteristics of IBD in our patients, the localization of disease, the biological behavior of the disease, and its treatment. Overall, including both adult and pediatric IBD, 9% of patients received biologics, with adalimumab biosimilars being prescribed more often than infliximab. It is likely that biologics will become more widely prescribed in IBD as more choices become available in this country. Interestingly, although societal guidelines de‐emphasize a role for mesalamine in the treatment of IBD, the finding that mesalamine was the most widely prescribed drug among all forms of IBD, probably illustrates the “real world experience” of benefit with mesalamine treatment.

The limitations of the present study include its cross‐sectional nature of data collection and a possible referral bias, which could have had an impact on the overall profile of IBD patients.

## Conclusion

The present study is a multi‐center survey from a single state in India to assemble epidemiologically relevant cross‐sectional data from more than a 1000 patients diagnosed with IBD across diverse age groups. This study is useful because it provides a snapshot of pediatric IBD in comparison with adult‐onset IBD at this particular point in time in India. This information will be useful in future comparisons as the epidemiology of IBD changes. The database available here can also be used as a resource for researchers in IBD who may be able to pursue specific research studies through the individual patient's caregiver. Continuing maintenance of the registry will enable a continuous epidemiological surveillance of IBD.
